# A Second Residual Tooth Occurring from One Tooth

**DOI:** 10.3390/diagnostics15060733

**Published:** 2025-03-14

**Authors:** Tatsuya Akitomo, Mariko Kametani, Yuko Iwamoto, Chieko Mitsuhata, Ryota Nomura

**Affiliations:** Department of Pediatric Dentistry, Graduate School of Biomedical and Health Sciences, Hiroshima University, Hiroshima 734-8553, Japan; mrysk25@hiroshima-u.ac.jp (M.K.); yuko-tulip@hiroshima-u.ac.jp (Y.I.); chiekom@hiroshima-u.ac.jp (C.M.); rnomura@hiroshima-u.ac.jp (R.N.)

**Keywords:** residual tooth, primary tooth, dental trauma

## Abstract

In clinical pediatric dentistry, dental professionals may encounter some dental abnormalities. It may progress in various ways; therefore, long-term follow-up is essential. We describe the case of a 5-month-old male, and how the mandibular incisor was lost due to trauma. He was referred to our hospital two days later, but the calcified tissue was detected in the oral cavity, confirming the residual tooth. We extracted the tooth, and a regular dental checkup revealed another residual tooth in the same region one year later. A residual tooth is considered to be affected by the Hertwig’s epithelial sheath, and it may occur when the root is immature. It is important for dental professionals to share this information and to continue long-term follow-up when they encounter patients at young ages who have had such teeth extracted or lost due to trauma.

**Figure 1:**
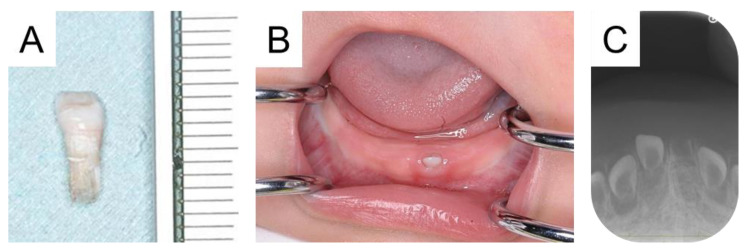
The first residual tooth occurring in the mandibular left primary central incisor. A 5-month-old male was referred to our hospital with a chief complaint of early tooth exfoliation. His mandibular left primary central incisor erupted at the age of 4 months, and it fell out due to trauma 2 days earlier. At the first visit, we confirmed the exfoliated tooth measuring 4.0 mm × 8.5 mm (**A**). However, our first visit detected a tooth-like structure in the region, which had mobility (**B**). The patient and his siblings had hypothyroidism, and he started medication of Levothyroxine Sodium Hydrate 11 days from birth due to high thyroid stimulating hormone levels, but there was no other medical nor family history. A radiographic examination did not reveal the tooth crown, which was diagnosed as a residual tooth (**C**). In addition, the tooth germ of another primary tooth was detected.

**Figure 2:**
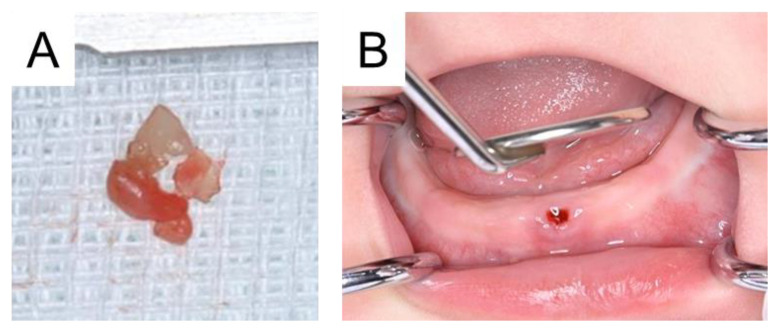
Photographs after tooth extraction. It was soft and had poor prognosis, and a tooth extraction was performed on the day (**A**). Intraoral examination confirmed no remaining residual teeth and hemostasis (**B**). Two months later, a mandibular right primary central incisor erupted in the oral cavity, and we continued the follow-up.

**Figure 3:**
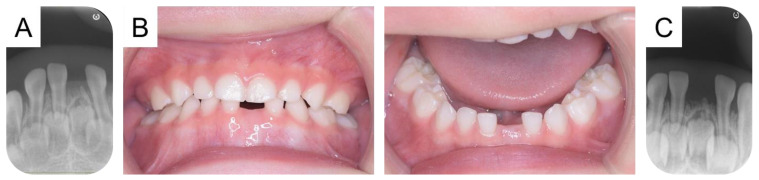
Intraoral and radiographic photographs during the follow-up period. We continued the patient’s follow-up and detected root-like tissue in the mandibular left primary central incisor region in a radiographic examination at 1 year and 5 months, confirming the diagnosis as another residual tooth (**A**). The patient was 2 years and 5 months, and 18 primary teeth erupted in the oral cavity, with no tooth crown in the residual tooth region. Additionally, no space loss or inclination of adjacent teeth was detected (**B**). The periapical photograph confirmed the root formation of the second residual tooth prog. We continued the long-term follow-up of the residual tooth (**C**). Tsubone et al. (2002) reported that a calcified structure was found in the oral cavity of a young girl, having erupted following the exfoliation of a natal tooth, and they termed this structure a residual natal tooth [1]. Natal teeth are defined as being present at birth and usually occur in pairs [2,3]. In the present case, the mandibular left primary central incisor erupted at 4 months and did not meet the definition of natal teeth. In addition, he had hypothyroidism, and the common oral features include delayed eruption of teeth [4]. In the present case, no delayed eruption of primary teeth was observed, and the influence of the systemic disease was ruled out. However, a residual tooth can occur in the same region not only in a natal tooth, but also in an immature permanent tooth or supernumerary teeth [5,6]. In addition, it is considered that the residual tooth is formed from the remaining Hertwig’s epithelial sheath derived from the extracted tooth [5,6]. This report highlights the possibility of a residual tooth when a tooth is extracted or exfoliated due to trauma before the root completion. In the present case, he has not yet experienced any cosmetic or masticatory effects from the absence of a tooth crown. However, children without anterior teeth are at risk of developing abnormal tongue habits or pronunciation disorders [7,8]. Regular radiographic examinations are conducted to check the residual tooth, and oral functions, including pronunciation or chewing, are also continually monitored. A limitation of this study is that the histopathological findings of the extracted tooth were not performed. Future studies including histopathological findings are needed.

## Data Availability

Data are contained within the article.
